# Associated factors and mediating effects on traditional Chinese medicine health information literacy in undergraduate nursing students

**DOI:** 10.3389/fpubh.2025.1570934

**Published:** 2025-06-18

**Authors:** Yanfei Liu, Jianke Lei, Luxin Wang, Zhenyuan Liu, Yu Wang, Hongxia Wang, Lamei Liu, Weihong Zhang

**Affiliations:** ^1^School of Nursing and Health, Zhengzhou University, Zhengzhou, China; ^2^Department of Cardiology, Henan Province Hospital of TCM, Zhengzhou, China; ^3^School of International Education, Zhengzhou University, Zhengzhou, China; ^4^Nursing Department, Henan Province Hospital of TCM, Zhengzhou, China; ^5^Henan Institute of Medical and Pharmaceutical Sciences, Zhengzhou University, Zhengzhou, China

**Keywords:** traditional Chinese medicine, health information literacy, nursing students, autonomous learning competency, associated factor

## Abstract

**Objective:**

To investigate the current status and associated factors of health information literacy in Traditional Chinese Medicine (TCM) among undergraduate nursing students and explore the mediating effects of these factors, with the aim of providing empirical evidence for optimizing the reform plan of TCM nursing curriculum.

**Methods:**

From November 2023 to December 2024, 215 undergraduate nursing students were selected as the research subjects and investigated using a general information questionnaire, the Health information literacy scale in TCM, and the scale of autonomous learning competencies of nursing undergraduates.

**Results:**

The overall scores of health information literacy in TCM of undergraduate nursing students was (122.68 ± 33.41). Among the average scores of each dimension, the scores for information morality and cognitive ability of health preserving information in TCM were relatively high, while the scores for retrieving ability of health preserving information in TCM, evaluation ability of health preserving information in TCM and application ability of health preserving information in TCM were in the lower range. The factors associated with health information literacy in TCM were the students’ place of residence, autonomous learning competency, and having access to reliable TCM health information (*p* < 0.05), which collectively explained 85.7% of the total variance. Both recognition and attention to TCM, as well as having access to reliable TCM health information, played a mediating role between the students’ place of residence and their health information literacy in TCM (*p* < 0.05).

**Conclusion:**

The health information literacy in TCM of undergraduate nursing students is associated with multiple factors, particularly their place of residence, autonomous learning competencies, and the availability of reliable health information in TCM. Universities should strengthen the cultivation of nursing undergraduates’ abilities to obtain and evaluate health information and optimize educational strategies for TCM nursing.

## 1 Introduction

Health information literacy refers to the ability of individuals or groups to recognize their health needs, acquire relevant information through appropriate channels, critically evaluate its applicability, and make informed health decisions (1, 2). The National Health and Family Planning Commission of China emphasized the ability to evaluate health information in the Chinese Citizens’ Health Literacy—Basic Knowledge and Skills (2024 Edition). It stated that health information literacy should encompass individuals’ ability to acquire, comprehend, evaluate, and apply health information (3). Health information literacy is a key indicator for assessing the health status of a population. Individuals with higher health information literacy are more proactive in seeking health information and are more likely to adopt appropriate health behaviors, which contributes to improving individual health outcomes and enhancing healthcare efficiency (4). TCM plays an irreplaceable role in disease prevention, treatment, and health maintenance. Assessing individuals’ health information literacy in TCM can effectively promote public health (5). Zhang et al. (2) designed a scale of health information literacy in TCM and validated its applicability for assessing the health information literacy of community residents through reliability and validity analysis. The study suggested that the health information literacy in TCM includes five dimensions: cognitive ability, acquisition ability, evaluation ability, application ability, and information ethics (2).

As an important breakthrough in enhancing health information literacy, the health information literacy in TCM is an important indicator of college students’ ability to judge the authenticity and validity of health information and assess its effectiveness, as well as an important way to promote TCM health culture and popularize TCM health care methods. Undergraduate nursing students’ TCM health awareness and ability to obtain information play an important role in enhancing patients’ health information literacy level (6, 7). However, a survey of college students’ health information literacy in TCM from TCM colleges and universities showed (8) that the overall level of college students’ health information literacy in TCM was 50.2%, which was lower than that of the older adult group or some special groups that need rehabilitation training (8, 9). At the same time, students with different characteristics showed significant differences in health information literacy levels, such as students with different characteristics of different genders, monthly household income, and whether or not to pay attention to TCM and its related knowledge acquisition methods showed significant differences in health information literacy levels, which seriously constrained the improvement of health information literacy among students (10, 11). This study aims to understand the current situation of health information literacy in TCM among undergraduate nursing students (hereinafter referred to as undergraduate nursing students) and to analyze the related factors in terms of demographic characteristics and autonomous learning competencies, with a view to providing a scientific basis for universities to improve the teaching reform strategy of TCM nursing.

Autonomous learning refers to students’ ability to effectively acquire and master the knowledge and skills essential for nursing practice by utilizing cognitive strategies and available human and material resources (12). According to Bandura’s social cognitive theory, the most important way to enhance self-efficacy is through successful experiences gained from autonomous learning, such as mastering new skills or solving complex problems. This “mastery experience,” as a direct success experience, has a greater impact than indirect observation or verbal persuasion (13). In the context of chronic disease management, individuals with strong autonomous learning competencies are better at utilizing health information to solve problems. Patients with high autonomous learning competencies are able to more effectively acquire and apply health information, thereby improving their disease management level (4, 14). In TCM education and outreach, stimulating individuals’ autonomous learning motivation helps update professional knowledge, enhances their ability to actively explore health information, and promotes the application of learned knowledge in health management, thereby improving the practical value of TCM education (15–17).

In summary, health information literacy in TCM includes the ability to acquire, analyze, evaluate, understand, and apply health information, as well as attitudes toward the use of health information (1, 2). Previous research on health information literacy has mostly focused on patients and community populations. As the future workforce for the development of TCM nursing and the promotion of TCM knowledge, the health information literacy in TCM and its associated factors among undergraduate nursing students still require further investigation (3). This study aims to understand the current status of health information literacy in TCM among undergraduate nursing students, analyze related factors such as demographic characteristics and autonomous learning competency, and based on the findings, explore effective measures to improve their health information literacy, in order to provide scientific evidence for the improvement of TCM nursing education reform strategies in universities.

## 2 Methods

### 2.1 Participants

Undergraduate nursing students from a comprehensive undergraduate college in Zhengzhou City, Henan Province, China, from November 2023 to January 2024, were selected for the study. Inclusion criteria: (1) full-time undergraduate nursing students; (2) informed consent and signing an informed consent form. Exclusion criteria: (1) Individuals who were on leave, including sick leave, personal leave, or any other form of leave, during the investigation period; (2) Individuals with mental disorders. This study was approved by the Ethics Committee of Zhengzhou University Ethics Committee for Life Sciences. Participants were informed of the study’s purpose and their voluntary participation. Anonymity was ensured by using coded identifiers and maintaining confidentiality in the handling of data. No financial compensation was provided for participation in this study.

### 2.2 Instruments

#### 2.2.1 General information questionnaire

The questionnaire included questions on gender, academic year, monthly household income, place of residence, whether the respondent or their family members had suffered from chronic diseases, their recognition and attention to TCM, their access to reliable TCM health information, whether family members were engaged in TCM-related work, and whether the participant had sought TCM treatment.

#### 2.2.2 Health information literacy scale in TCM

It was compiled by Zhang et al. (2) and his research team in 2019 to evaluate the community citizens’ health information literacy in TCM. The scale consists of 5 domains covering 36 items, including the cognitive ability of health preserving information in TCM (5 items), retrieving ability of health preserving information in TCM (11 items), evaluation ability of health preserving information in TCM (11 items), application ability of health preserving information in TCM (7 items) and information morality (2 items). Using the Likert 5-point scale, responses range from “Strongly Disagree” (scored as 1) to “Strongly Agree” (scored as 5), with the total score ranging from 36 to 180. A higher score indicates a higher level of an individual’s health information literacy in TCM. The scale demonstrated strong content validity, with item-level content validity indices ranging from 0.79 to 1.00 and a scale-level content validity index of 0.91, exceeding the recommended threshold of 0.80. Regarding construct validity, the Kaiser-Meyer-Olkin (KMO) value of the pilot version was 0.858, and Bartlett’s test of sphericity was significant (χ^2^ = 4432.38, *p* < 0.001), indicating that the data were suitable for factor analysis. Principal component analysis with eigenvalues greater than 1.00 extracted five components, explaining 65.29% of the total variance. These components were identified as: (1) cognitive ability of TCM health-preserving information, (2) retrieval ability, (3) evaluation ability, (4) application ability, and (5) information morality, with factor loading ranging from 0.42 to 0.86. The internal consistency of the scale was excellent, with a Cronbach’s alpha coefficient of 0.981. Although initially validated in community populations (2), subsequent studies have confirmed its applicability and psychometric robustness among nursing students (18).

#### 2.2.3 Scale of autonomous learning competencies of nursing undergraduates

It was developed by Lin and Jiang (19) and his research team in 2004, and was used to assess the level of nursing students’ autonomous learning competency. The scale encompasses three dimensions: autonomous management competency (10 items), informative competency (11 items), and cooperative learning competency (7 items), totaling 28 items. It adopts a Likert 5-point scaling method, where responses range from “Strongly Disagree” to “Strongly Agree,” assigned values from 1 to 5, respectively. For items 1, 2, 5, 10, 11, 13, and 27, “Strongly Agree” is scored as 1 point, and “Strongly Disagree” is scored as 5 points. The total score ranges from 28 to 140, with higher scores indicating stronger autonomous learning competency among nursing students. The Cronbach’s alpha coefficient of the scale was 0.984.

### 2.3 Sample size calculation

According to the overall mean estimation sample size calculation formula, after literature review (2), the mean score of the health information literacy in TCM was found to be 127, with a standard deviation (*σ*) of 15, and a tolerable error (*δ*) of 2, at a significance level (*α*) of 0.05. Considering a 20% quality issue (or allowing for a 20% margin of error due to potential quality issues), the final result indicated that the required sample size was at least 190 cases.

### 2.4 Data collection

To protect the privacy of the respondents, the questionnaires were completed independently and anonymously. The investigator used uniform instruction to explain to the respondents the purpose and significance of the survey, the method of completing the questionnaire, and the precautions to be taken. The questionnaires were distributed and collected on the spot. For questionnaires that could not be collected on the spot, the researcher agreed with the respondents on a time and place to collect them within the agreed period. A total of 230 questionnaires were distributed and 215 valid questionnaires were recovered, with a valid questionnaire recovery rate of 93.47 percent.

### 2.5 Statistical analysis

SPSS 26.0 software was used for statistical analysis; count data were described by frequency and percentage; measured data were described by mean and standard deviation; the correlation between the health information literacy in TCM and autonomous learning competency was analyzed by Pearson correlation analysis, and multiple linear regression was used to analyze the relevant factors of TCM. Mediation analyses were computed via SPSS PROCESS Model 4, with the number of Bootstrap sampling iterations set to 5,000. Statistically significant differences were indicated by *p* < 0.05.

## 3 Results

### 3.1 The results of univariate analysis of health information literacy in TCM scores of undergraduate nursing students with different characteristics

A total of 215 undergraduate nursing students were included in this study. Among them, 49 (22.8%) were male and 166 (77.2%) were female. Additionally, 40 (18.6%) were first-year students, 45 (20.9%) were second-year students, 43 (20.0%) were third-year students, and 87 (40.5%) were fourth-year students. Statistically significant differences (*p* < 0.05) were found in the scores of health information literacy in TCM based on gender, academic year, monthly household income, the place of residence, whether they or their family members had chronic diseases, their recognition and attention to TCM, their access to reliable sources of TCM health information, whether they had family members engaged in TCM-related work, and their experience of TCM treatment. See Table 1 for details.

**Table 1:** Univariate analysis of health information literacy (HIN) in TCM of undergraduate nursing students with different characteristics (*n* = 215).

Characteristics	HIN in TCM scores (Mean ± SD)	*t*/*F*-value	*p*-value
Gender		−2.975	0.003
Men	108.46 ± 35.5		
Women	126.88 ± 31.68		
Academic year		32.513	<0.001
First-year	103.42 ± 40.41		
Second-year	96.37 ± 29.39		
Third-year	136.16 ± 24.66		
Fourth-year	138.49 ± 20.5		
Household income (RMB/month)		24.645	<0.001
<1,000	120.37 ± 33.3		
1,000–1999	99.45 ± 30.16		
2000–2,999	130.54 ± 28.66		
3,000–4,999	145.51 ± 18.67		
≥5,000	151.93 ± 14.23		
Place of residence		9.421	<0.001
Urban	142.47 ± 23.02		
Rural	107.02 ± 32.07		
Chronic disease history (self/family)		3.690	0.026
Yes	133.87 ± 29.25		
No	103.03 ± 31.27		
Recognition and attention to TCM		12.759	<0.001
Yes	141.20 ± 20.10		
No	94.36 ± 29.69		
Access to reliable health information literacy in TCM		14.437	<0.001
Yes	144.12 ± 19.42		
No	96.11 ± 27.52		
Family engagement in TCM-related work		12.242	<0.001
Yes	150.20 ± 13.72		
No	111.53 ± 32.59		
Sought TCM treatment		11.058	<0.001
Yes	142.47 ± 20.79		
No	101.96 ± 31.56		

SD, standard deviation; RMB, renminbi; HIN, health information literacy.

### 3.2 Scores of undergraduate nursing students on health information literacy in TCM and autonomous learning competencies

The total score for health information literacy in TCM among undergraduate nursing students was (122.68 ± 33.41), indicating a middle level of proficiency. Similarly, the total score for autonomous learning competency was (91.48 ± 29.79), also reflecting a middle level. Please refer to Table 2 for detailed information.

**Table 2:** Scores of undergraduate nursing students on health information literacy in TCM and autonomous learning competencies (*n* = 215).

Categories	Theoretical range	Observed range	Mean ± SD	95% CI
Health information literacy in TCM	36–180	54–180	122.68 ± 33.41	118.22–127.16
Cognitive ability	5–25	5–25	18.43 ± 5.64	17.68–19.19
Retrieving ability	11–55	11–55	37.52 ± 11.17	36.03–39.01
Evaluation ability	11–55	11–55	34.11 ± 11.73	32.54–35.68
Application ability	7–35	9–35	23.95 ± 6.30	23.11–24.80
Information morality	2–10	3–10	8.66 ± 1.43	8.47–8.86
Autonomous learning competency	28–140	28–140	91.48 ± 29.79	87.51–95.47
management competency	10–50	10–50	32.63 ± 11.56	31.09–34.18
Informative competency	11–55	11–55	36.10 ± 11.00	34.64–37.58
Cooperative competency	7–35	7–35	22.74 ± 7.93	21.69–23.81

SD, standard deviation; CI, confidence interval; Theoretical range, score range calculated based on the scale design; Observed range, actual scores collected in this study.

### 3.3 Multi-factorial analysis of undergraduate nursing students’ health information literacy scores in TCM

Factors that were statistically significant in the univariate analysis were used as independent variables, while the total score of health information literacy in TCM was used as the dependent variable. Multiple linear regression was performed to analyze these variables, and the variable assignments are shown in Table 3. The results indicated that place of residence, access to reliable TCM health information, and autonomous learning competency were contributing factors to the level of health information literacy in TCM among undergraduate nursing students (*p* < 0.05). The adjusted *R*^2^ value was 0.857, indicating that the independent variables in this regression equation explain 85.7% of the variation in health information literacy levels. Hypothesis testing results of the regression model showed that *F* = 126.655, with a *p* < 0.001, indicating that the regression equation is statistically significant. The Variance Inflation Factor (VIF) values of the independent variables were all less than 5, suggesting insignificant multicollinearity. Please refer to Table 4 for detailed information.

**Table 3:** Coding methods for independent variables in the analysis.

Independent variable	Assignment method
Gender	0 = Male; 1 = Female
Academic year	1 = First-year; 2 = Second-year; 3 = Third-year; 4 = Fourth-year
Household income (RMB/month)	1 = <1,000; 2 = 1,000–1999; 3 = 2000–2,999; 4 = 3,000–4,999; 5 = ≥5,000
Place of residence	0 = Rural; 1 = Urban
Chronic disease history (self/family)	0 = No; 1 = Yes
Recognition and attention to TCM	0 = No; 1 = Yes
Access to reliable health information literacy in TCM	0 = No; 1 = Yes
Family in TCM-related work	0 = No; 1 = Yes
Sought TCM treatment	0 = No; 1 = Yes
Total score of autonomous learning competency	Continuous (original scores)

**Table 4:** Multiple linear regression analysis of factors associated with TCM health information literacy scores among undergraduate nursing students (*n* = 215).

Predictors	*β*	SE	Standardized *β*	*t-value*	*p*-value	VIF
Constant	14.702	7.594	–	1.720		
Place of residence (Ref: rural)	5.338	2.633	0.080	2.027	0.044	2.263
Having access to reliable TCM health information (Ref: No)	14.195	2.741	0.212	5.178	<0.001	2.458
Total score of autonomous learning competency (total score)	0.999	0.056	0.891	18.136	<0.001	3.550

*β*, unstandardized regression coefficient; SE, standard error; VIF, variance inflation factor; Ref, reference category.

### 3.4 Mediating effects of health information literacy in TCM among undergraduate nursing students

“Recognition and attention to TCM,” “Access to reliable TCM health information,” and “Family in TCM-related work” were all predictors of health information literacy in TCM (*p* < 0.001). Among these, “Access to reliable TCM health information” had the highest explanatory power for health information literacy in TCM, with *R*^2^ = 0.267, *F* = 77.522, and *t* = 8.805 (*p* < 0.001). Please refer to Table 5 for detailed information. “Recognition and attention to TCM” and “Access to reliable TCM health information” mediated the relationship between place of residence and the level of health information literacy in TCM (*p* < 0.05), with mediation effect values of 9.016 and 12.773, respectively. The 95% confidence intervals (CIs) for the total, direct, and indirect effects of both mediating models did not contain 0. The mediating effect of “Place of residence,” “Family in TCM-related work” was not significant. Please refer to Table 6 for detailed information.

**Table 5:** Mediation analysis of health information literacy in TCM among undergraduate nursing students.

Variables	Fitting index		Coefficient significance	
	*R^2^*	*F-*value	*t*	*p-*value
Recognition and attention to TCM	0.171	43.794	6.618	<0.001
Access to reliable health information literacy in TCM	0.267	77.522	8.805	<0.001
Family in TCM-related work	0.326	102.896	10.144	<0.001

**Table 6:** Mediating effect sizes of health information literacy in TCM among undergraduate nursing students.

Model path	Indirect effect	95% CI	SE	Conclusion
Place of residence → Recognize and care about TCM → Health information literacy in TCM	9.016	0.070–0.211	0.036	Partial mediation
Place of residence → Having access to reliable TCM health information → Health information literacy in TCM	12.773	0.109–0.275	0.043	Partial mediation
Place of residence → Family in TCM-related work → Health information literacy in TCM	2.884	0.041–0.114	0.039	The mediating effect was not significant.

SE, standard error; CI, bias-corrected bootstrap confidence interval (5,000 samples).

## 4 Discussion

### 4.1 The current status of health information literacy in TCM among undergraduate nursing students

#### 4.1.1 The health information literacy in TCM among undergraduate nursing students was considered to be at a medium level

The health information literacy in TCM, not only reflects an individual nursing undergraduate student’s mastery of health knowledge and its acquisition pathways but also places emphasis on evaluating the individual’s capabilities in applying and assessing health information. In this study, the total score for the health information literacy in TCM among undergraduate nursing students was 122.68 ± 33.41, which is considered to be at a medium level. It is noteworthy that the higher the grade level of undergraduate nursing students, the higher their health information literacy scores. The reason for this may be that the relevant courses on “TCM Nursing” in Western medicine-oriented institutions are mainly offered in the second and third years. These courses cover the basic theories and knowledge of TCM, which help nursing students better understand TCM health culture and further enhance their recognition and acceptance of TCM (20). Furthermore, the progressive accumulation of professional knowledge with academic advancement appears to foster stronger health awareness among undergraduate nursing students (21). Previous researches on TCM nursing education conducted in universities had indicated that the blended teaching strategies, integrating both online and offline modalities, may be particularly effective in enhancing students’ health information literacy in TCM (22, 23). For instance, offline practical sessions in TCM health exercises, supplemented by online thematic lectures and virtual simulations of TCM nursing techniques, can stimulate students’ learning motivation and strengthen their professional competencies in TCM nursing (22, 23). Moreover, collaborations with TCM clinics to provide practical training opportunities can facilitate case-based learning and mentor-guided practice, thereby promoting the seamless integration of theoretical knowledge with clinical application. Early immersion in the cultural dimensions of TCM nursing through such educational models not only reinforce the identification with TCM principles but also contributes to the overall improvement of health information literacy among undergraduate nursing students (23–25).

#### 4.1.2 Improvement needed in the evaluation and application ability of health information literacy in TCM among undergraduate nursing students

This study indicates that the majority of nursing students have a relatively low level of evaluation and application ability regarding health information, which is consistent with the findings of Guo et al. (18). On the one hand, this could be due to the limited opportunities for nursing students to engage in TCM nursing practice and to have clinical clerkships in TCM departments during their school years, resulting in a low level of proficiency in applying TCM health preservation techniques (26). On the other hand, it may also be attributed to the wide variety and varying quality of relevant online information, as well as insufficient training in online health information literacy skills provided by universities (27, 28). Therefore, nursing educators should integrate TCM culture into teaching practices in combination with professional characteristics, achieving a seamless integration of theory and practice. They should improve practical teaching conditions, standardize the practical teaching curriculum system, and increase the proportion of practical courses to effectively enhance nursing students’ clinical practical abilities in TCM nursing. Secondly, universities should strengthen training in health information literacy in TCM, focusing on cultivating students’ abilities to evaluate and judge health information, thereby improving their overall level of health information literacy in TCM.

### 4.2 Associated factors and mediating effects related to health information literacy in TCM among undergraduate nursing students with different characteristics

#### 4.2.1 The significant role of reliable access to health information in TCM among undergraduate nursing students

In this study, 44.6% of nursing students believed that the health information sources they obtained on TCM health care were unreliable. In contrast, undergraduate nursing students who had access to reliable health information sources on TCM health care demonstrated higher health information literacy in TCM (*p* < 0.05). This finding is consistent with the results of the study conducted by Zhao et al. (29). This may be attributed to the fact that although most nursing students are able to locate and learn about health information through the Internet, they lack the ability to assess the accuracy and truthfulness of the information, and their ability to apply health information is relatively insufficient. This reflects the characteristic of “high perception, low behavior” (30). Therefore, universities should enhance students’ ability to evaluate and utilize health information in TCM by improving the teaching content of TCM nursing, recommending professional TCM information resource platforms to students, and incorporating information literacy education as well as relevant practical courses.

#### 4.2.2 The level of health information literacy in TCM shows a clear urban–rural gap

This study found that undergraduate nursing students from urban areas had significantly higher levels of TCM health information literacy than those from rural areas (*p* < 0.05). One potential explanation is that 80% of urban students reported access to reliable TCM health information sources, compared to only 32.5% of rural students. Mediation analysis further revealed that place of residence significantly influenced TCM health information literacy through “recognition of and attention to TCM” and “access to reliable TCM information sources” (*p* < 0.05), suggesting that urban students benefit more resource availability (31, 32).

However, the variable “family members engaged in TCM-related work” did not demonstrate a significant mediating effect. Several factors may explain this unexpected finding. First, from a methodological perspective, the operationalization of this variable focused solely on occupational ties, rather than direct familial practices or household health behaviors, which may have underestimated its potential influence (33). Second, from a developmental perspective, undergraduate students may exhibit greater autonomy in health decision-making, thus diminishing the direct impact of familial occupational background and highlighting the more prominent role of formal education in shaping health information literacy (34). This result contrasts with findings in older adult populations, where family involvement in TCM-related work has been significantly associated with higher levels of TCM health literacy (33). Such discrepancies may indicate a generational shift in the pathways of cultural knowledge transmission, with younger cohorts increasingly relying on structured educational experiences rather than traditional familial influence. Collectively, these results underscore the importance of strengthening undergraduate nursing students’ recognition and engagement with TCM through targeted educational initiatives. Future research could employ mixed-methods approaches to further explore generational differences in cultural transmission and their implications for the development of TCM health information literacy.

#### 4.2.3 Positive correlation between autonomous learning ability and health information literacy in TCM

The stronger the autonomous learning competencies of the undergraduate nursing students in this study, the higher their level of health information literacy in TCM. The reason for this correlation was analyzed: health information literacy is a long-term, or even lifelong, process of training and improvement (35). Undergraduate nursing students who scored higher on autonomous learning demonstrated a greater ability to self-manage, explore information, and collaborate in learning. When encountering difficulties, they were able to effectively use information media to identify health information needs, screen sources of health information, and continuously enhance their health information literacy (36). Therefore, it is crucial for undergraduate nursing students to improve their autonomous learning competencies. Nursing teachers can utilize a variety of teaching strategies and methods, such as the Internet cooperative learning method, the peer support learning model, and simulated clinical situations, to guide nursing students in establishing a correct concept of independent learning and to stimulate their interest in learning (37, 38). At the same time, they should pay greater attention to the core professional qualities of nursing students (10, 38, 39). The educators can combine the theoretical model of core literacy of college students in TCM to further clarify the objectives of professional training, optimize the education and teaching process, and cultivate TCM nursing talents suitable for future needs.

#### 4.2.4 TCM course availability and cultural factors associated with health literacy

The institutional TCM curriculum design may partially explain the observed variations in health literacy outcomes. Prior studies have indicated that students completing three or more TCM-related courses achieved significantly higher scores in TCM health literacy focused on health preservation compared to those who only completed a single course (40). This finding aligns with our results showing that reliable access to TCM information correlates strongly with enhanced health literacy (*p* < 0.05). While TCM cultural literacy encompasses multidimensional capabilities including information acquisition, comprehension, evaluation, and practical application, existing curricula often demonstrate insufficient depth and an unbalanced emphasis across these dimensions, thereby limiting overall literacy development (34, 41). To address these limitations, TCM nursing education reform should simultaneously pursue curricular restructuring to strengthen core competency integration and establish standardized assessment protocols incorporating cultural cognitive adaptability and critical information evaluation metrics. This dual approach would create an integrated competency-curriculum-assessment framework to achieve structural literacy improvements.

### 4.3 Limitations

While this study offers meaningful insights into health information literacy among TCM nursing students, several limitations warrant acknowledgement. First, the single-institution design in Henan province may limit generalizability due to regional particularities in both cultural attitudes toward TCM and curriculum implementation. For instance, students with more than 3 years of residence in inland China demonstrated stronger confidence in TCM nursing development and a higher willingness to participate in TCM training programs (42). Additionally, variations in curriculum design such as course content and structure, may serve as important institutional determinants of TCM health information literacy (34). Second, the cross-sectional design precludes definitive causal inferences. Future research should employ multicenter designs encompassing regions with varying levels of TCM policy support and curriculum development, and longitudinal follow-up to better identify causal pathways and key determinants of TCM health literacy.

## 5 Conclusion

The health information literacy in TCM among undergraduate nursing students was found to be at a moderate level. This literacy is associated with multiple factors, particularly their place of residence, autonomous learning competencies, and the accessibility of reliable health information in TCM. Universities should enhance the cultivation of nursing undergraduates’ abilities to obtain and evaluate health information and optimize educational strategies for TCM nursing. This study provides references for the implementation of health literacy education in TCM, aiming to enhance the TCM health literacy levels among undergraduate students and to inform curriculum reform and innovation strategies.

## Data Availability

The raw data supporting the conclusions of this article will be made available by the authors, without undue reservation.
